# Choosing not to look: not a solution

**DOI:** 10.1093/eurpub/ckac113

**Published:** 2022-08-25

**Authors:** Rosemary Frasso

**Affiliations:** College of Population Health, Thomas Jefferson University, Philadelphia, PA, USA

*‘just have a heart…please don’t look at us like we’re garbage’*,

(Philadelphia man, explaining what he would like a passerby to do).

Poverty remains a fundamental driver of mental and physical health, as well as of other human conditions that matter to those of us in public health. While the number of people living in poverty around the world had been declining, the COVID-19 pandemic caused irreparable damage to the global economy, thereby reversing this trend.^1^ Poverty is complicated; operational definitions have varied across time, location and circumstance. The presence or absence of escape routes out of poverty is variable, too—confounded by important social justice issues, including, classism, racism, sexism, ableism and biases against gender and sexual minorities. Drug traffic, exposure to violence and the physical and sexual exploitation of the world’s most vulnerable exacerbates the brutal conditions inflicted by poverty. Both political instability and the climate crisis will continue to force people from their homes and nations—therein worsening global access to housing, food, water and sustaining employment.

While there is a great deal of public health literature evaluating anti-poverty programs, we know very little about the lived experience of those in need, especially relating to their interactions with others in shared spaces.

Panhandling—the act of asking for help from those who are passing by—is one pretext by which the housed and unhoused in our society interact. Not everyone who is unhoused panhandles and not all panhandlers are unhoused. However, panhandling or begging, is most certainly a consequence of some substantial need. A symptom of a brutally unequal society, panhandling takes on many forms; it looks different around the world. In many US cities, handmade signs are common. Some people will engage passersby in conversation, explaining quickly what they need, and while in many European cities one often sees people sitting, kneeling or praying behind a cup, silently asking or hoping for help. In South America, I have seen children darting through traffic offering to wash car windshields in exchange for change. There are far too many examples to describe, each illustrative of the ways in which we do engage with one another, even in nations and cities that are designed to silo us, and place the violence of poverty behind smokescreens. Our recent work in Philadelphia focused on these inevitable interactions.^2^ In particular, we spoke to people who use cardboard signs to ask for money or in-kind donations.^2^

We learned quickly, not to anyone’s surprise, that the experience of panhandling is humiliating, exceedingly stressful, isolating and lonely. While panhandling is a highly visible crisis in modern cities, individuals who panhandle can and do feel unseen. The stress caused by the social isolation may worsen pre-existing mental and physical illness in a population already plagued by such ailments.^3^ Studies have found that unhoused people face incessant stigma and rejection when trying to engage with people within and outside of their community, and that negative interactions with people who do not share their experience, namely those who have never had to panhandle, can further contribute to feelings of neglect among those who do, negatively impacting their well-being.^3^^,^^4^

In the USA, a misguided movement, aimed at reducing the discomfort felt by those who wish to ‘ignore’ the plight of poverty, has taken hold in many cities: anti-homeless architecture, sometimes termed ‘excluding design’. Many park benches, retaining walls, shaded areas and entry ways, are now fitted with barriers or fixtures that make it impossible to lie down, sit or shelter from the sun, wind, rain or snow.^5^ These horrific designs are heavy-handed metaphors for society’s broader neglect of people who are poor. They prioritize the feelings of those with privilege—to be shielded from viewing the disastrous consequences of American wealth inequality—over the material needs of those who are e struggling with poverty, perhaps compounded by a substance use disorder or a mental health crisis. We are not going to solve these material issues or other social injustices by moving human beings ‘out of sight’; we need to face our discomfort, and while we work toward sustainable solutions, we need to increase and promote human interaction between those in need and those who pass them by. This does not necessarily mean giving money to everyone you see; it does mean we need to recognize the humans in front of us, in all moments, regardless of their conditions and of ours.

We have failed to measure the impact of these interactions on those in need, so we do not know how much negative exchanges, including being completely ignored, may further deepen an already stark divide. Perhaps such interactions make it harder for people who panhandle to seek support, find help or engage in programs or public health interventions. While poverty must be alleviated through broadscale re-configurations of our resources as organized societies, we cannot discount the impact or value of human interactions and we should work to improve them, too.

*Conflicts of interest*: None declared.
